# Plasma neutrophil gelatinase-associated lipocalin predicts acute kidney injury, morbidity and mortality after pediatric cardiac surgery: a prospective uncontrolled cohort study

**DOI:** 10.1186/cc6192

**Published:** 2007-12-10

**Authors:** Catherine L Dent, Qing Ma, Sudha Dastrala, Michael Bennett, Mark M Mitsnefes, Jonathan Barasch, Prasad Devarajan

**Affiliations:** 1 Department of Cardiology, Cincinnati Children's Hospital Medical Center, University of Cincinnati School of Medicine, 3333 Burnet Ave, Cincinnati, Ohio 45229, USA; 2 Department of Nephrology & Hypertension, Cincinnati Children's Hospital Medical Center, University of Cincinnati School of Medicine, 3333 Burnet Ave, Cincinnati, Ohio 45229, USA; 3 Department of Nephrology, College of Physicians and Surgeons, Columbia University, 630 West 168^th ^Street, New York, New York 10032, USA

## Abstract

**Introduction:**

Acute kidney injury (AKI) is a frequent complication of cardiopulmonary bypass (CPB). The lack of early biomarkers has impaired our ability to intervene in a timely manner. We previously showed in a small cohort of patients that plasma neutrophil gelatinase-associated lipocalin (NGAL), measured using a research enzyme-linked immunosorbent assay, is an early predictive biomarker of AKI after CPB. In this study we tested whether a point-of-care NGAL device can predict AKI after CPB in a larger cohort.

**Methods:**

First, in a cross-sectional pilot study including 40 plasma samples (NGAL range 60 to 730 ng/ml) and 12 calibration standards (NGAL range 0 to 1,925 ng/ml), NGAL measurements by enzyme-linked immunosorbent assay and by Triage^® ^NGAL Device (Biosite Inc., San Diego, CA, USA) were highly correlated (*r *= 0.94). Second, in a subsequent prospective uncontrolled cohort study, 120 children undergoing CPB were enrolled. Plasma was collected at baseline and at frequent intervals for 24 hours after CPB, and analyzed for NGAL using the Triage^® ^NGAL device. The primary outcome was AKI, which was defined as a 50% or greater increase in serum creatinine.

**Results:**

AKI developed in 45 patients (37%), but the diagnosis using serum creatinine was delayed by 2 to 3 days after CPB. In contrast, mean plasma NGAL levels increased threefold within 2 hours of CPB and remained significantly elevated for the duration of the study. By multivariate analysis, plasma NGAL at 2 hours after CPB was the most powerful independent predictor of AKI (β = 0.004, *P *< 0.0001). For the 2-hour plasma NGAL measurement, the area under the curve was 0.96, sensitivity was 0.84, and specificity was 0.94 for prediction of AKI using a cut-off value of 150 ng/ml. The 2 hour postoperative plasma NGAL levels strongly correlated with change in creatinine (*r *= 0.46, *P *< 0.001), duration of AKI (*r *= 0.57, *P *< 0.001), and length of hospital stay (*r *= 0.44, *P *< 0.001). The 12-hour plasma NGAL strongly correlated with mortality (*r *= 0.48, *P *= 0.004) and all measures of morbidity mentioned above.

**Conclusion:**

Accurate measurements of plasma NGAL are obtained using the point-of-care Triage^® ^NGAL device. Plasma NGAL is an early predictive biomarker of AKI, morbidity, and mortality after pediatric CPB.

## Introduction

Cardiopulmonary bypass (CPB) surgery is the most frequent major surgical procedure performed in hospitals worldwide, with well over a million operations undertaken each year in adults alone [1]. Acute kidney injury (AKI), previously referred to as acute renal failure, is a frequent and serious complication encountered in 30% to 50% of subjects after CPB [2,3]. AKI requiring dialysis occurs in up to 5% of these cases, in whom the mortality rate approaches 80%, and is the strongest independent risk factor for death with an odds ratio of 7.9 [4]. Even minor degrees of postoperative AKI, as manifest by only a 0.2 to 0.3 mg/dl rise in serum creatinine from baseline, predict a significant increase in short-term mortality [5,6]. AKI after cardiac surgery is also associated with a number of adverse outcomes, including prolonged intensive care and hospital stay, dialysis dependency, diminished quality of life, and increased long-term mortality [7-9].

Clinical investigations have identified several risk factors associated with the development of AKI after CPB, the majority related to either impaired renal perfusion or decreased renal reserve, and have resulted in the development of clinical scoring systems for the prediction of AKI [10-13]. However, these tools have not been validated across medical centers and have focused primarily on identifying the small number of high-risk, dialysis-requiring patients. Concomitant advances in the basic sciences have illuminated the pathogenesis of AKI and have paved the way to successful therapeutic approaches in animal models [14]. However, translational research efforts in humans have yielded disappointing results, and no corresponding preventive or therapeutic strategy has been successful [2,15]. A major reason for the failure to find an effective treatment in patients is the paucity of early biomarkers for AKI, akin to troponins in acute myocardial disease, and hence a delay in initiating therapy [16]. In current clinical practice, the 'gold standard' for identification and classification of AKI is dependent on serial serum creatinine measurements [17], which are especially unreliable during acute changes in kidney function [15,16].

We utilized a genome-wide interrogation strategy to identify kidney genes that are induced very early after AKI in animal models, whose protein products might serve as novel early biomarkers. We identified neutrophil gelatinase-associated lipocalin (NGAL) as one of the most upregulated genes in the kidney soon after ischemic injury [18-20]. NGAL protein was also markedly induced in kidney tubule cells, and easily detected in the plasma and urine in animal models of ischemic and nephrotoxic AKI [18-22]. The expression of NGAL protein was also dramatically increased in kidney tubules of humans with ischemic, septic, and post-transplant AKI [23,24]. Importantly, NGAL in the plasma was found to be an early predictive biomarker of AKI in a variety of acute clinical settings in pilot studies [25]. In a cohort of 20 patients who developed AKI 2 to 3 days after cardiac surgery, plasma NGAL measured using a research enzyme-linked immunosorbent assay (ELISA) was elevated within 2 to 6 hours after CPB [16]. Preliminary results using the research-based assay also suggest that plasma NGAL measurements predict AKI after contrast administration [26]. The availability of a validated point-of-care tool for NGAL measurements could revolutionize renal diagnostics in critical care situations [27]. Therefore, the first objective of the present study was to determine whether a rapid, standardized point-of-care NGAL assay correlates with the research-based assay. The second objective was to determine the utility of the point-of-care NGAL assay as a predictive biomarker of AKI after CPB in a large prospective pediatric cohort.

## Materials and methods

### Patients and study design

This investigation was approved by the institutional review board of the Cincinnati Children's Hospital Medical Center. All children undergoing elective CPB for surgical correction or palliation of congenital heart lesions between January 2004 and June 2006 were prospectively enrolled. We obtained written informed consent from the legal guardian of every participant before enrolment. Exclusion criteria included pre-existing renal insufficiency, diabetes mellitus, peripheral vascular disease, and use of nephrotoxic drugs before or during the study period.

To obviate postoperative volume depletion and prerenal azotemia, all patients received at least 80% of their maintenance fluid requirements during the first 24 hours after surgery and 100% maintenance subsequently. We obtained spot plasma samples at baseline and at frequent intervals (2, 6, 12, and 24 hours) after initiation of CPB. Samples were stored at -80°C. Serum creatinine was measured by the hospital clinical laboratory at baseline and routinely monitored at least twice daily during the first 2 days after CPB, and at least daily after the third postoperative day.

The primary outcome variable was the development of AKI, defined as a 50% or greater increase in serum creatinine from baseline. This corresponds to the risk phase of the RIFLE (risk, injury, failure, loss, and end-stage kidney) criteria for diagnosis of AKI [17]. Other outcomes included percentage change in serum creatinine, days in AKI, dialysis requirement, length of hospital stay, and mortality. Other variables we obtained included age, sex, ethnic origin, CPB time, previous heart surgery, and urine output.

In a pilot cross-sectional study, we measured NGAL concentrations in 40 plasma samples and 11 calibration standards to determine the correlation between the two assay methods described below. In a subsequent prospective study, serial plasma samples from 120 children undergoing CPB were assayed for NGAL using the Triage^® ^device (Biosite Inc., San Diego, CA, USA) to assess its ability to predict AKI and other adverse outcomes.

### NGAL analysis using the Triage^® ^point-of-care device

The Triage^® ^NGAL test is a point-of-care, fluorescence-based immunoassay used in conjunction with the Triage Meter (Biosite Inc.) for the rapid quantitative measurement of NGAL concentration in EDTA-anticoagulated whole blood or plasma specimens. The assay device is a single-use plastic cartridge that contains an NGAL-specific monoclonal antibody conjugated to a fluorescent nanoparticle, NGAL antigen immobilized on a solid phase, and stabilizers. In addition, the device is engineered with integrated control features including positive and negative control immunoassays, which ensure that the test performs properly and that the reagents are functional. The test is performed by inoculating several drops of whole blood or plasma into the sample port where the specimen moves through an integrated filter to separate cells from plasma. The plasma then reconstitutes the fluorescent antibody conjugate detection nanoparticles and flows down the diagnostic lane via capillary action. NGAL present in the specimen prevents binding of the fluorescent detection particles to the solid phase immobilized in the detection zone, such that the analyte concentration is inversely proportional to the fluorescence detected. Separate solid phase zones are located along the same diagnostic lane for the control assay systems. The device is then inserted into the Triage Meter, a portable fluorescence spectrometer, and quantitative measurements of NGAL concentration in the range from 60 to 1,300 ng/ml are displayed on the meter screen and/or printout in approximately 15 minutes. Calibration information is relayed to the meter via a lot-specific EPROM chip (the code chip module).

### NGAL analysis by ELISA

The plasma NGAL ELISA was performed using an established and validated assay as previously described [16,26]. Briefly, microtiter plates precoated with a mouse monoclonal antibody raised against human NGAL (#HYB211-05; AntibodyShop, Gentofte, Denmark) were blocked with buffer containing 1% bovine serum albumin, coated with 100 μl of samples (plasma) or standards (NGAL concentrations ranging from 1 to 1,000 ng/ml), and incubated with a biotinylated monoclonal antibody against human NGAL (#HYB211-01B; AntibodyShop) followed by avidin-conjugated horseradish peroxidase (Dako, Carpinteria, California, USA). TMB substrate (BD Biosciences, San Jose, California, USA) was added for color development, which was read after 30 minutes at 450 nm with a microplate reader (Benchmark Plus; Bio-Rad, Hercules, CA, USA). All measurements were made in triplicate. Precoated plates can be refrigerated and used for several days, and the entire ELISA procedure is typically completed in 4 hours. The inter- and intra-assay coefficient variations were under 5% for batched samples analyzed on the same day, and under 10% for the same sample measured 6 months apart. The laboratory investigators were blinded to the sample sources and clinical outcomes until the end of the study.

### Statistical analysis

Statistical analysis was performed using SAS version 9.2 (SAS Institute Inc., Cary, NC, USA). Either a two-sample *t*-test or Mann-Whitney rank sum test was used for continuous variables, whereas χ^2 ^or Fisher's exact test was used for categorical variables. The associations between variables were assessed by Spearman rank order correlation analysis. Univariate and multivariate stepwise regression analyses were undertaken to assess predictors of AKI after CPB. Potential independent predictor variables included age, sex, ethnicity, CPB time, and history of prior cardiac surgery. To calculate the sensitivity and specificity for the plasma NGAL measurements at varying cut-off values, a conventional receiver operating characteristic curve was generated and the area under the curve (AUC) was calculated to quantify the accuracy of plasma NGAL as a biomarker. An AUC of 0.5 is no better than expected by chance, whereas a value of 1.0 signifies a perfect biomarker. *P *≤ 0.05 was considered statistically significant.

## Results

### Verification of the Triage^® ^point-of-care NGAL device

The Triage^® ^NGAL test was found to have a minimum detectable NGAL concentration of 60 ng/ml and an upper limit of detection of 1,300 ng/ml, and exhibited a linear response to NGAL concentration over this range. The average within-day coefficient of variance was 11%, with a total precision of 14% when assessed over 20 consecutive days at three NGAL levels spread across the reportable range. Biologically relevant levels of hemoglobin, triolein, bilirubin, and rheumatoid factors did not interfere with the recovery of NGAL. Commonly used pharmaceuticals and contrast agents tested at therapeutically relevant concentrations also did not interfere with the Triage^® ^NGAL Test (Triage^® ^NGAL product insert; Biosite Inc.).

**Figure 1:**
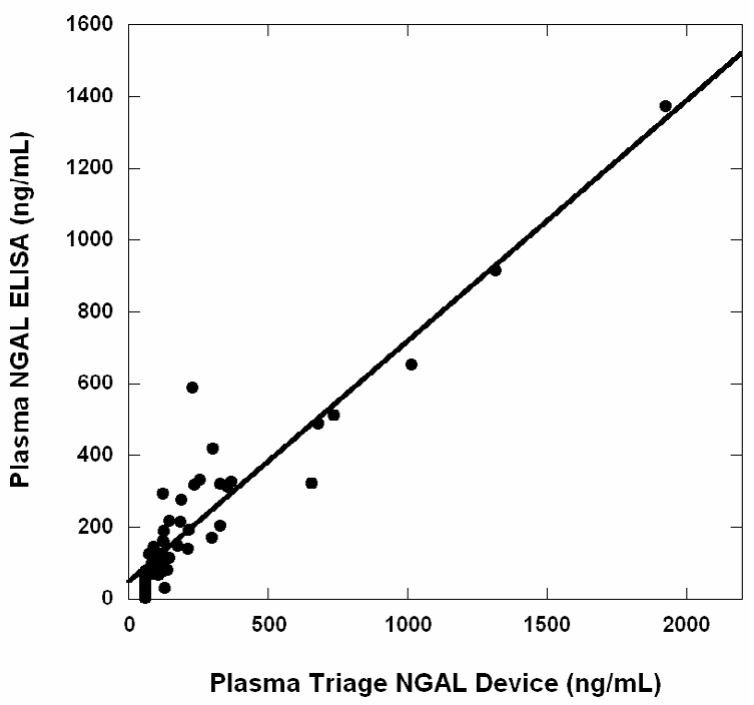
Correlation between Triage^® ^NGAL device and ELISA. Shown is the correlation between plasma NGAL measurements obtained by Triage^® ^NGAL device and research-based NGAL ELISA assay (Pearson *r *= 0.94, 95% confidence interval 0.89 to 0.96; *P *< 0.001). The regression line shown yielded a slope of 0.671 (95% confidence interval 0.600 to 0.741) and an intercept of 48.82 (95% confidence interval 18.66 to 78.99). ELISA, enzyme-linked immunosorbent assay; NGAL, neutrophil gelatinase-associated lipocalin.

The cross-sectional phase of this study was designed to verify the Triage^® ^NGAL device against the research-based NGAL ELISA assay. As shown in Figure 1, NGAL concentrations in 40 random plasma samples from patients undergoing CPB (NGAL range 60 to 730 ng/ml) and 11 calibration standards (NGAL range 0 to 1,925 ng/ml) determined using the two assays were highly correlated (Pearson *r *= 0.94, 95% confidence interval 0.89 to 0.96; *P *< 0.001). From a linear regression analysis, the observed slope was 0.671 (95% confidence interval 0.600 to 0.741) with an intercept of 48.82 (95% confidence interval 18.66 to 78.99). The slight deviation from unity observed between the two methods in this correlation analysis probably arose from differences in NGAL concentration assignments for the samples used to calibrate these two assays.

### NGAL as a predictor of acute kidney injury and other adverse outcomes

In a subsequent prospective study, serial plasma samples from 120 children who met the inclusion and exclusion criteria were assayed for NGAL using the Triage^® ^device to assess its ability to predict AKI and other adverse outcomes. Forty-five patients (37%) met the criteria for AKI within a 3-day period. However, the increase in serum creatinine by 50% or greater from baseline was delayed by 2 to 3 days after CPB. Based on this primary outcome, we classified patients into those with and those without AKI. No differences were noted with respect to age, sex, or race (Table 1). All patients received a similar postoperative fluid regimen, and there were no differences in the volume status or urine output between the two groups.

**Table 1:** Patient characteristics, clinical outcomes, and plasma NGAL measurements

Parameter	No AKI (*n *= 75)	AKI (*n *= 45)	*P*
Age (years)	3.4 ± 0.5	4.9 ± 0.7	NS
Males (%)	55	50	NS
Caucasians (%)	85	88	NS
Prior surgery (%)	39	55	0.01
Bypass time (min)	99 ± 5.4	143 ± 9.0	<0.0001
Creatinine change (%)	11 ± 1.5	117 ± 19	<0.0001
Duration of AKI (days)	0	3 ± 0.7	<0.0001
Hospital stay (days)	5.8 ± 0.7	12.7 ± 1.6	<0.0001
Deaths (%)	0	16	<0.0001
Plasma NGAL baseline (ng/ml)	66.1 ± 2.0	75.5 ± 3.1	0.07
Plasma NGAL 2 hours (ng/ml)	84.1 ± 4.2	218.8 ± 12.1	<0.0001
Plasma NGAL 12 hours (ng/ml)	68.4 ± 2.2	219.1 ± 22.0	<0.0001
Plasma NGAL 24 hours (ng/ml)	72.9 ± 5.9	232.6 ± 41.2	<0.0001

Values are expressed as means ± standard deviation. AKI, acute kidney injury; NGAL, neutrophil gelatinase-associated lipocalin; NS, not signfiicant.

In patients that developed AKI, the duration of CPB was significantly longer, and the clinical outcomes were significantly worse. The serum creatinine rose by a greater percentage in the AKI group, and both length of hospitalization and mortality rate were significantly higher (Table 1). Among patients with AKI, two (4.5%) required dialysis, primarily for fluid overload. There were a total of seven deaths, all in the AKI group. The causes of death were multiorgan failure in five and sepsis in two patients.

**Figure 2:**
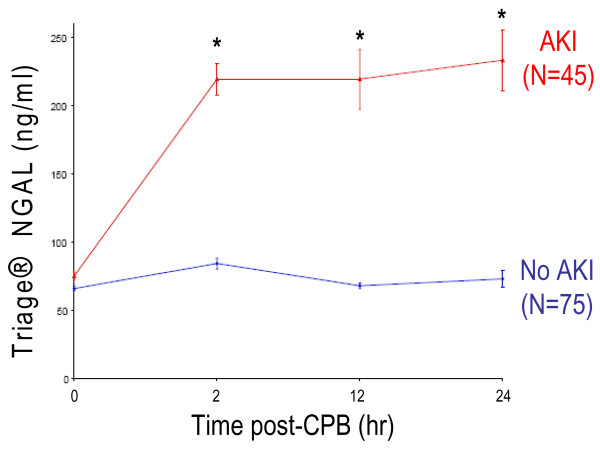
Plasma NGAL measurements obtained using Triage^® ^NGAL device at various time points after CPB. AKI was defined as a 50% increase in serum creatinine from baseline. Values are expressed as means ± standard deviation. *P < 0.0001 comparing AKI versus no AKI groups. AKI, acute kidney injury; CPB, cardiopulmonary bypass; NGAL, neutrophil gelatinase-associated lipocalin.

Plasma NGAL measurements at baseline were comparable in the AKI and non-AKI groups (Table 1). In the non-AKI group there was a small but statistically significant increase in plasma NGAL at 2 hours after CPB, which normalized back to baseline levels at the 12-hour and 24-hour time points. In marked contrast, in patients who subsequently developed AKI there was a robust threefold increase in plasma NGAL at 2 hours after CPB, which persisted at the 12-hour and 24-hour time points (Figure 2).

To assess independent predictors for the development of AKI in the entire cohort, multivariate logistic regression was performed. All variables that were found by univariate analysis to display a *P *< 0.1 were entered into the model. Plasma NGAL measurement at 2 hours after CPB was the most powerful independent predictor of AKI (β = 0.004, *P *< 0.0001). Other predictors of AKI included history of previous cardiac surgery (*β *= 0.22, *P *= 0.003) and CPB time (β = 0.001, *P *= 0.03), yielding a model *R*^2 ^= 0.64. Age, sex, and race were not independent predictors of AKI.

To test the hypothesis that plasma NGAL levels measured soon after CPB could be used to predict eventual clinical outcomes, a Spearman rank order correlation analysis was performed. The 2-hour NGAL levels strongly correlated with percentage change in serum creatinine (*r *= 0.46, *P *< 0.001), duration of AKI (*r *= 0.57, *P *< 0.001), and length of hospital stay (*r *= 0.44, *P *< 0.001). The 12-hour NGAL levels strongly correlated with mortality (*r *= 0.48, *P *= 0.004) as well as all of the measures of morbidity mentioned above.

**Figure 3:**
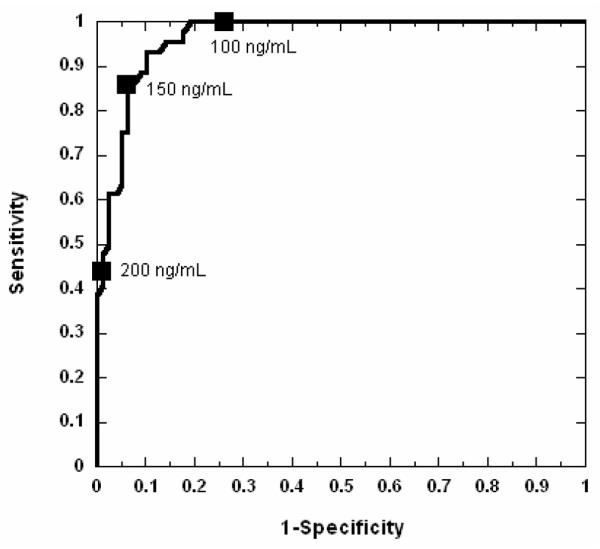
ROC analysis of 2-hour NGAL at three cut-offs. Shown is a ROC curve analysis of the 2-hour plasma NGAL measurements with the three cut-off levels from Table 2 indicated as filled squares annotated with the corresponding NGAL concentration. The area under the curve was 0.96 (95% confidence interval 0.94 to 0.99). NGAL, neutrophil gelatinase-associated lipocalin; ROS, receiver operating characteristic.

To assess the utility of NGAL measurements at varying cut-off values to predict AKI, a conventional receiver operating characteristic curve was generated and the AUC calculated. Table 2 lists the derived sensitivities, specificities, and predictive values at different cut-off concentrations. For plasma NGAL at 2 hours after CPB, sensitivity and specificity were optimal at the 150 ng/ml cut-off, with an AUC of 0.96 (95% confidence interval 0.94 to 0.99) for the prediction of AKI (Figure 3).

**Table 2:** Plasma NGAL test characteristics at various cut-off values for the 2-hour time point

	Cut-off point (ng/ml)		
	
	≥ 200	≥ 150	≥ 100
Sensitivity (%)	43	84	100
Specificity (%)	99	94	75
Positive predictive value (%)	44	84	100
Negative predictive value (%)	98	93	74

NGAL, neutrophil gelatinase-associated lipocalin.

## Discussion

Serum creatinine is an inadequate marker for AKI [25]. First, more than 50% of renal function must be lost before an elevation in serum creatinine is detected. Second, serum creatinine does not accurately depict kidney function until a steady state has been reached, which may require several days. Although animal studies have shown that AKI can be prevented and/or treated using several maneuvers, these must be instituted very early after the insult, well before the rise in serum creatinine becomes apparent. Our study indicates that monitoring of plasma NGAL levels can potentially provide a very early warning to providers of critical care. The 2-hour plasma NGAL level measured using the Triage^® ^NGAL device was an excellent biomarker for the subsequent development of AKI and its complications. The assay is facile and performed on the Triage Meter with quantitative results available within approximately 15 minutes, and requires only microliter quantities of whole blood or plasma. The assay is autocalibrated and includes reactive internal controls that run with every sample applied. It has been suggested that a clinically acceptable assay for diagnosing AKI should be a robust system that can measure the appropriate analyte rapidly day or night [28]. The Triage NGAL test provides quantitative NGAL measurements in minutes and is deployable directly to the point of patient care, and thus satisfies these requirements. Furthermore, the Triage Meter and test devices for cardiac markers have been adopted by clinical institutions world wide, providing further evidence that this system is robust.

Human NGAL, a member of the lipocalin superfamily, was initially described as a 25 kDa protein that is covalently bound to gelatinase in neutrophils and expressed at low concentrations in normal kidney, trachea, lungs, stomach, and colon [29]. NGAL expression is induced in injured epithelia, including lung, colon, and especially kidney [18-25]. Emerging experimental and clinical evidence indicates that in the early phases of AKI from diverse etiologies, NGAL accumulates within two distinct pools, namely a systemic and a renal pool. It has been demonstrated that AKI results in increased NGAL mRNA expression in distant organs, especially the liver and spleen, and the over-expressed NGAL protein is most likely released into the circulation and constitutes the systemic pool [30,31]. Additional contributions to the systemic pool in AKI may derive from the fact that NGAL is a known acute phase reactant and may be released from neutrophils, macrophages, and other immune cells [32]. Furthermore, any decrease in glomerular filtration rate resulting from AKI would be expected to decrease the clearance of NGAL, with further accumulation in the systemic pool. Gene expression studies in AKI have also shown rapid upregulation of NGAL mRNA in the thick ascending limb of Henle's loop and the collecting ducts, with resultant synthesis of NGAL protein in the distal nephron (the renal pool) and secretion into the urine where it comprises the major fraction of urinary NGAL [30,31].

This study lends support to our previous findings in a small cohort of 20 patients who developed AKI 2 to 3 days after cardiac surgery, in whom plasma NGAL measured by a research ELISA was elevated within 2 to 6 hours following CPB [16]. In both the previous and the present study, patients who developed AKI also encountered a longer CPB time, raising the possibility that plasma NGAL levels reflected the duration of CPB rather than kidney injury. A larger randomized controlled trial will be required to determine whether plasma NGAL levels truly predict AKI or whether they merely reflect longer CPB times. However, subsequent studies have demonstrated that the utility of plasma NGAL measurements is not restricted only to the CPB population. For example, plasma NGAL is also an early, sensitive, specific, and predictive biomarker of AKI after contrast administration [26].

Our study has several strengths. First, we prospectively recruited a relatively homogeneous cohort of pediatric patients in whom the only obvious etiology for AKI would be the result of CPB. These patients comprise an ideal and important population for the study of AKI biomarkers, because they do not exhibit common comorbid variables that complicate similar studies in adults, such as diabetes, hypertension, atherosclerosis, and nephrotoxin use [33]. Second, all patients started with normal kidney function, and the study design allowed for the precise temporal definition of altered plasma NGAL concentrations and a direct comparison with subsequent changes in serum creatinine. Our results clearly indicate that plasma NGAL is a powerful early biomarker of AKI that precedes the increase in serum creatinine by several hours to days. The magnitude of rise supports the notion that plasma NGAL is a highly discriminatory biomarker with a wide dynamic range and cut-off values that allow for early risk stratification. Third, this is the first example of how a standardized point-of-care platform may be useful for predicting AKI using plasma samples in critical care settings. The majority of biomarkers of AKI described thus far have been measured in the urine [25]. Urinary diagnostics do have several advantages, including the noninvasive nature of sample collection, the reduced number of interfering proteins, and the potential for the development of self-testing kits. However, several disadvantages also exist, including the lack of sample from patients with severe oliguria, and potential changes in urinary biomarker concentration induced by hydration status and diuretic therapy. Plasma-based diagnostics have revolutionized many facets of critical care, as exemplified by the use of troponins for the early diagnosis of acute myocardial infarction and the value of B-type natriuretic peptide for prognostication in acute coronary syndrome.

This study has important limitations. First, it is a single-center uncontrolled cohort study of pediatric patients with congenital heart defects undergoing elective CPB. Our results, although provocative and of clear statistical significance, will certainly need to be validated in a larger randomized prospective trial, including adults with the usual confounding variables and comorbid conditions that normally accumulate with increasing age. Until the appropriate studies are completed, the results cannot be extrapolated to the adult CPB population. In addition, a larger randomized controlled trial in children will be required to determine whether plasma NGAL levels truly predict AKI or whether they merely reflect longer CPB times. Second, ours was a cohort with normal kidney function at recruitment, and it will be important to confirm our findings in documented high-risk settings such as pre-existing kidney dysfunction, diabetes mellitus, and concomitant nephrotoxic drug use. Third, other potential confounding variables that could lead to AKI in this population, such as inotrope support and complexity of surgery, were not considered in the multivariate analysis. It is likely that these parameters contributed to the AKI in our cohort, in addition to the duration of CPB. Fourth, in addition to NGAL, simultaneous examination of other plasma and urinary biomarkers as potential predictors of AKI may be informative [25]. It is likely that not one single biomarker such as NGAL but rather a collection of strategically selected candidates will provide the hitherto elusive panel for early and rapid diagnosis of AKI.

In the critical care setting, an early elevation in plasma NGAL would trigger immediate intervention. At the very least, clinicians informed of such a situation would avoid the use of additional nephrotoxins, and optimize hydration and renal perfusion to prevent further injury. The ability to predict which patients will develop AKI after CPB may also add substantively to existing clinical scoring systems, and enable early initiation of interventions to change the dismal outcomes associated with this all too common clinical problem.

## Conclusion

AKI is a frequent and serious complication after CPB. The paucity of early biomarkers for AKI, akin to troponins in acute myocardial disease, has crippled our ability to initiate timely therapy in the critical care setting. In this study, we have shown that rapid and reliable measurements of plasma NGAL are obtained using the newly developed point-of-care Triage^® ^NGAL device, and that plasma NGAL is an early predictive biomarker of AKI, morbidity, and mortality after pediatric CPB.

## Key messages

• Rapid and reliable measurements of plasma NGAL are obtained using the newly developed point-of-care Triage^® ^NGAL device.

• Whereas the diagnosis of AKI using serum creatinine was delayed by 2 to 3 days, mean plasma NGAL levels increased threefold within 2 hours of CPB.

• Plasma NGAL at 2 hours after CPB was the most powerful independent predictor of AKI.

• For the 2-hour plasma NGAL measurement, the AUC was 0.96, sensitivity was 0.84, and specificity was 0.94 for prediction of AKI using a cut-off value of 150 ng/ml.

• The 2-hour plasma NGAL levels strongly correlated with severity and duration of AKI, and length of hospital stay. In addition, the 12-hour plasma NGAL strongly correlated with mortality.

## Abbreviations

AKI = acute kidney injury; AUC = area under the curve; CPB = cardiopulmonary bypass; ELISA = enzyme-linked immunosorbent assay; NGAL = neutrophil gelatinase-associated lipocalin.

## Competing interests

Biosite^® ^Incorporated has signed an exclusive licensing agreement with Cincinnati Children's Hospital and Columbia University for developing plasma NGAL as a biomarker of acute renal failure. Dr Devarajan has received honoraria for speaking engagements from Biosite^® ^Incorporated.

## Authors' contributions

CLD, JB and PD had the idea for and designed the study, and recruited the patients. QM and SD processed the samples and performed all the laboratory assays. MMM and PD performed the statistical analyses. All authors contributed to data interpretation and writing of the manuscript.
